# Supplementary material for: Soluble and multivalent Jag1 DNA origami nanopatterns activate Notch without pulling force
Source: Nat Commun. 2024 Jan 18;15:465. doi: 10.1038/s41467-023-44059-4 (PMC10796381; doi:10.1038/s41467-023-44059-4)
Supplement: Supplementary file 3 — Reporting Summary [file 41467_2023_44059_MOESM3_ESM.pdf]

nature portfolio

Corresponding author(s): Björn Högberg

Last updated by author(s): Nov 22, 2023

Reporting Summary

Nature Portfolio wishes to improve the reproducibility of the work that we publish. This form provides structure for consistency and transparency in reporting. For further information on Nature Portfolio policies, see our Editorial Policies and the Editorial Policy Checklist.

Statistics

For all statistical analyses, confirm that the following items are present in the figure legend, table legend, main text, or Methods section.

n/a	Confirmed	
☐	☒	The exact sample size (*n*) for each experimental group/condition, given as a discrete number and unit of measurement
☐	☒	A statement on whether measurements were taken from distinct samples or whether the same sample was measured repeatedly
☐	☒	The statistical test(s) used AND whether they are one- or two-sided<br>*Only common tests should be described solely by name; describe more complex techniques in the Methods section.*
☒	☐	A description of all covariates tested
☐	☒	A description of any assumptions or corrections, such as tests of normality and adjustment for multiple comparisons
☐	☒	A full description of the statistical parameters including central tendency (e.g. means) or other basic estimates (e.g. regression coefficient) AND variation (e.g. standard deviation) or associated estimates of uncertainty (e.g. confidence intervals)
☐	☒	For null hypothesis testing, the test statistic (e.g. *F*, *t*, *r*) with confidence intervals, effect sizes, degrees of freedom and *P* value noted<br>*Give P values as exact values whenever suitable.*
☒	☐	For Bayesian analysis, information on the choice of priors and Markov chain Monte Carlo settings
☒	☐	For hierarchical and complex designs, identification of the appropriate level for tests and full reporting of outcomes
☒	☐	Estimates of effect sizes (e.g. Cohen's *d*, Pearson's *r*), indicating how they were calculated

Our web collection on statistics for biologists contains articles on many of the points above.

Software and code

Policy information about availability of computer code

Data collection	NextSeq550 illumina; Zeiss LSM980-Airy2; BIAevaluation 3.2 software (GE Healthcare);StepOne version 2.3; Talos 120V microscope; Nikon Eclipse Ti-E inverted microscope (Nikon Instruments)
Data analysis	Visualization and statistics were performed using GraphPad Prism v.8.0.2<br>During the RNA sequencing analysis, the Raw data was adapter trimmed and human transcriptome (GRCh38.p13 Gencode v38 protein-coding transcripts; gencode.v38.pc_transcripts) and quantified using Salmon(57) (v1.3.0, options: -l ISR --validateMappings). Quantifications were summarized to gene-level using tximport(58) (v1.12.3) and differential expression was calculated as Wald tests using DESeq2(59) (v1.24.0). Gene ontology enrichment was performed as Fisher overrepresentation tests using PANTHER(60) (release 20210224, GO database 10.5281/zenodo.5080993 and release 20221103, GO:0030100 Regulation of Endocytosis).<br>The Picasso Localize software was used to detect and fit localizations in the raw DNA PAINT movies using the MLE algorithm (Box sixe:7, Min. Net Gradient: 2000, EM Gain: 2, Baseline: 43.2, Sensitivity: 4.1, Quantum efficiency: 0.98, Pixel size: 87nm).

For manuscripts utilizing custom algorithms or software that are central to the research but not yet described in published literature, software must be made available to editors and reviewers. We strongly encourage code deposition in a community repository (e.g. GitHub). See the Nature Portfolio guidelines for submitting code & software for further information.

Data

Policy information about availability of data

All manuscripts must include a data availability statement. This statement should provide the following information, where applicable:

- Accession codes, unique identifiers, or web links for publicly available datasets
- A description of any restrictions on data availability
- For clinical datasets or third party data, please ensure that the statement adheres to our policy

The detailed DNA origami design schematics including sequences has been deposited at https://nanobase.org (accession 192, https://nanobase.org/structure/192 and accession 231 https://nanobase.org/structure/231)
Raw sequencing data have been deposited at ArrayExpress (accession E-MTAB-12439) and are publicly available as of the date of publication.
Other raw data is submitted as the Source Data File
Computational code for RNA-seq analyses and DNA-PAINT image analysis is available at https://github.com/bhogberg/nanoscale-notch-multivalency

Research involving human participants, their data, or biological material

Policy information about studies with human participants or human data. See also policy information about sex, gender (identity/presentation), and sexual orientation and race, ethnicity and racism.

Reporting on sex and gender	N/A
Reporting on race, ethnicity, or other socially relevant groupings	N/A
Population characteristics	N/A
Recruitment	N/A
Ethics oversight	N/A

Note that full information on the approval of the study protocol must also be provided in the manuscript.

Field-specific reporting

Please select the one below that is the best fit for your research. If you are not sure, read the appropriate sections before making your selection.

☒ Life sciences ☐ Behavioural & social sciences ☐ Ecological, evolutionary & environmental sciences

For a reference copy of the document with all sections, see nature.com/documents/nr-reporting-summary-flat.pdf

Life sciences study design

All studies must disclose on these points even when the disclosure is negative.

Sample size	No statistical sample size calculations were done prior to the experiments. The sample size was chosen based on the type of experiment and whether the chosen sample size gave reproducibility of the results in several independent experiments. When data were consistent across two (2), three (3) or four (4) independent experiments, we considered the sample size to be sufficient.
Data exclusions	No data was excluded
Replication	Most experiments are reproduced at least three times and all replicate data is shown.
Randomization	The cell experiments were not randomized because the cells used in the different experimentes were derived from the same cell population.
Blinding	For the proximity ligation assay experiment (PLA), analysis was conducted in a blinded format whereby knowledge of which conditions applied to which groups of cells held by one author was withheld from the author responsible for image acquisition and analysis to avoid bias.

Reporting for specific materials, systems and methods

We require information from authors about some types of materials, experimental systems and methods used in many studies. Here, indicate whether each material, system or method listed is relevant to your study. If you are not sure if a list item applies to your research, read the appropriate section before selecting a response.

Materials & experimental systems

n/a	Involved in the study
☐	☒ Antibodies
☐	☒ Eukaryotic cell lines
☒	☐ Palaeontology and archaeology
☒	☐ Animals and other organisms
☒	☐ Clinical data
☒	☐ Dual use research of concern
☒	☐ Plants

Methods

n/a	Involved in the study
☒	☐ ChIP-seq
☒	☐ Flow cytometry
☒	☐ MRI-based neuroimaging

Antibodies

Antibodies used

Rabbit IgG monoclonal antibody for the detection of cleaved Notch1 (Val1744) (D3B8) (Cell Signaling Technology, cat. no. 4147) ;
NOTCH1 Monoclonal Antibody (Thermo Fisher SCIENTIFIC, # MA5-11961);
Goat anti-Mouse IgG (H+L) Cross-Adsorbed Secondary Antibody, Alexa Fluor™ 488 (Thermo Fisher SCIENTIFIC, #A-11001);

Validation

All antibodies used in this study were chosen based on the validation statements for species (human or mouse) and application (WB, IP, ICC, IHC, or Flow) on the manufacturer's website.

Eukaryotic cell lines

Policy information about cell lines and Sex and Gender in Research

Cell line source(s)

lt-NES cells were obtained from the iPS Core facility at Karolinska Institutet.

Authentication

All cell lines were authenticated by morphology, growth and PCR assays.

Mycoplasma contamination

Cells were tested negative for mycoplasma

Commonly misidentified lines
(See ICLAC register)

No commonly misidentified cell lines were used in this study.
